# Musculoskeletal model of osseointegrated transfemoral amputees in OpenSim

**DOI:** 10.1371/journal.pone.0288864

**Published:** 2023-09-28

**Authors:** Vishal Raveendranathan, Vera G. M. Kooiman, Raffaella Carloni

**Affiliations:** 1 Bernoulli Institute for Mathematics, Computer Science and Artificial Intelligence, Faculty of Science and Engineering, University of Groningen, Groningen, The Netherlands; 2 Orthopaedic Research Laboratory and Department of Rehabilitation, Donders Institute for Brain, Cognition and Behavior, Radboud University Medical Center, Nijmegen, The Netherlands; Opole University of Technology: Politechnika Opolska, POLAND

## Abstract

This study presents a generic OpenSim musculoskeletal model of people with an osseointegrated unilateral transfemoral amputation wearing a generic prosthesis. The model, which consists of seventy-six musculotendon units and two ideal actuators at the knee and ankle joints of the prosthesis, is tested by designing an optimal control strategy that guarantees the tracking of experimental amputee data during level-ground walking while finding the actuators’ torques and minimizing the muscle forces. The model can be made subject-specific and, as such, is able to reproduce the kinematics and dynamics of both healthy and amputee subjects. The model provides a tool to analyze the biomechanics of level-ground walking and to understand the contribution of the muscles and of the prosthesis’ actuators. The proposed OpenSim musculoskeletal model is released as support material to this study.

## 1 Introduction

Musculoskeletal modeling is essential for the analysis of human locomotion as it is an instrumental tool to bring experimental data into a simulation environment and, therefore, to deeply understand pathological human gaits or plan efficient subject-specific rehabilitation strategies [1–4]. Steele *et al*. showed how subject-specific modeling can be used to improve the gait of children walking with a mild crouch. By using modeling and the insights gained from simulations, surgeries were performed to correct the gait and, as a consequence, to improve the patients’ quality of life [5]. Similarly, in [6], a musculoskeletal model of a subject walking with an instrumented knee implant was developed to understand the tibiofemoral mechanical loads during walking and, eventually, to find a novel method for the retraining of muscle coordination. Ranz *et al*. used musculoskeletal modeling for the analysis of limb alignment and for planning surgical transfemoral amputation techniques [7]. Harandi *et al*. developed a ten-segment model to understand compensatory gait mechanisms during overground walking [8], and to analyze individual muscle contribution to the mechanical loads between the hip joint and a bone-anchored implant [9]. Raveendranathan *et al*. developed a skeletal model to study the biomechanics of a transfemoral osseointegrated amputee [10].

**Fig 1:**
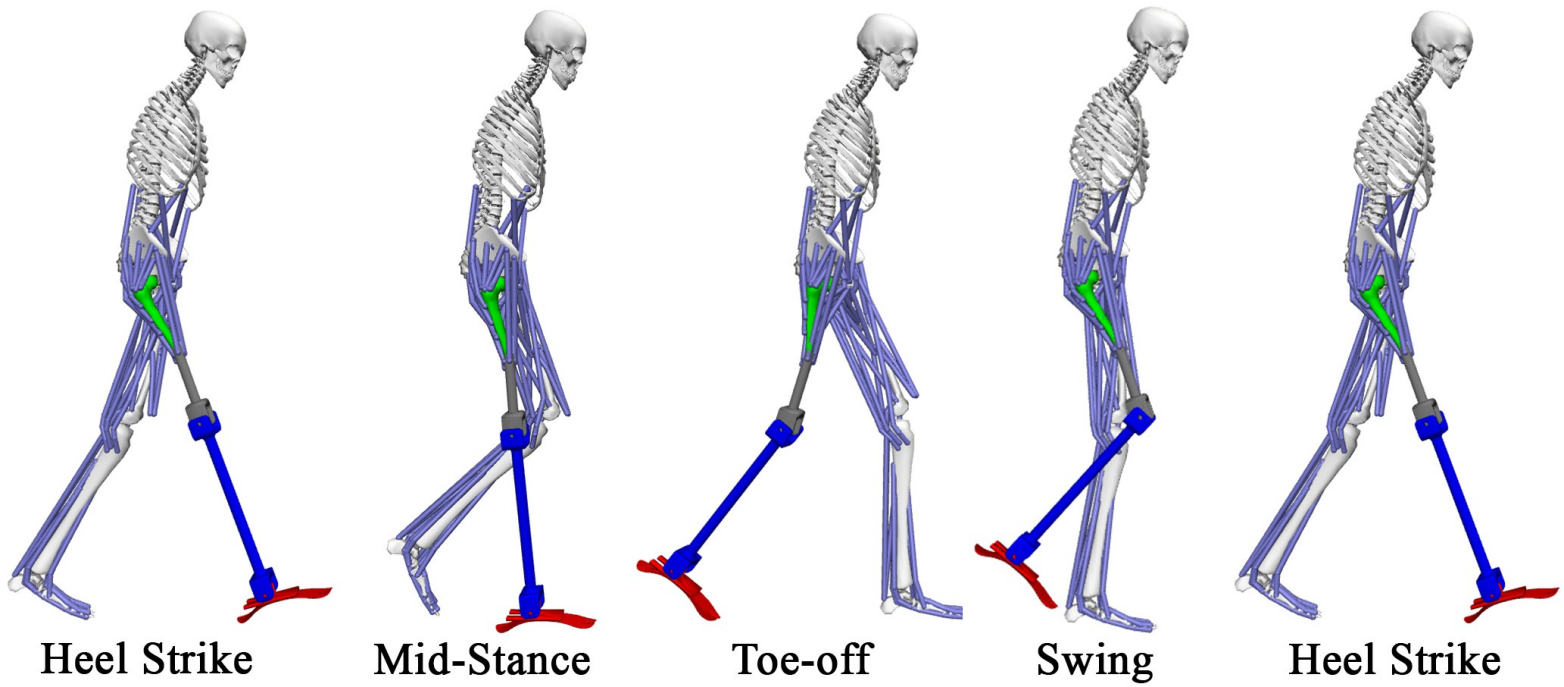
The proposed lower-extremity musculoskeletal model of people with an osseointegrated unilateral transfemoral amputation (of the right leg but without loss of generality) wearing a generic transfemoral prosthesis during level ground walking in the OpenSim simulation environment.

This study introduces a *novel generic lower-extremity musculoskeletal model of people with an osseointegrated unilateral transfemoral amputation wearing a generic transfemoral prosthesis*, as sketched in Fig 1 during one complete gait cycle. The model has been developed in the open-source modeling and simulation software OpenSim (NIH National Center for Simulation in Rehabilitation Research, Stanford, CA, USA, https://opensim.stanford.edu/), which has become the gold standard to perform simulations for biomechanical research [11–16]. Specifically, the model consists of 19 degrees of freedom, which are controlled by 76 musculotendon units and 2 ideal actuators at the knee and ankle joints of the prosthesis, and can be made subject-specific by adding the main physical characteristic of the patient and of the used prosthesis. The model has been developed with the main goal of providing the scientific community with a powerful tool that can be used to perform biomechanical analysis of osseointegrated subjects and to design control architecture for the used prosthesis. The proposed model extends the current literature that consists of an OpenSim musculoskeletal model of socket-user people with unilateral transtibial amputation wearing the open-source bionic leg [17], which has been tested via inverse kinematics analysis on experimental motion capture data [18].

This study introduces also a *novel optimal control architecture* that has been designed with the goal of performing the forward dynamic simulation of the proposed model during level ground walking. Inspired by the OpenSim research on exoskeletons [19, 20], the control architecture modifies and extends the computed muscle control of OpenSim to be able to simultaneously activate the musculotendon units of the model and to actuate the ideal actuators of the prosthesis. Specifically, thanks to this optimal control architecture, the model is able to track experimental amputee data during level-ground walking while minimizing the muscle forces and selecting the appropriate actuators’ torques.

The remainder of the paper is organized as follows. Section 2 describes the data collection and experimental protocol. Section 3 presents the novel generic musculoskeletal model of people with an osseointegrated transfemoral amputation wearing a generic prosthesis that has been developed in OpenSim. Section 4 describes the control architecture to perform the inverse and forward dynamic simulations of the proposed model. In Section 5, the results of the simulations performed on the model with data from two osseointegrated participants and one able-body participant during level-ground walking are shown and discussed. Finally, concluding remarks are drawn in Section 6.

## 2 Protocol and data collection

The data used for this study were collected at the Radboud University Medical Center in Nijmegen, The Netherlands. The study, under protocol number NL2018–4919, was evaluated and received a waiver by the Medical Ethics Review Committee Arnhem-Nijmegen (Nijmegen, The Netherlands) on December 10, 2018, and complied with the guidelines as defined in the Declaration of Helsinki [21]. Participants signed a written informed consent before participating.

### 2.1 Protocol

The participants were asked to walk on an instrumented treadmill for 5 minutes at their comfortable walking speed (Motek Medical B.V., Houten, The Netherlands, www.motekmedical.com). Ground reaction forces were collected by two embedded force plates, one for each foot, at a sampling rate of 2 kHz. 3D movements of the participants were captured by a Vicon motion capture system (Vicon Motion Systems Ltd, Oxford, United Kingdom, www.Vicon.com). The participants’ kinematic data were collected using ten Vicon cameras at 100 frames per second sampling rate. Reflective markers were placed on the participants’ skin according to the guidelines of the Vicon full-body plug-in-gait marker protocol [22]. Using the motion capture system, the raw marker positions and force plate data were exported as a .*c3d* file.

### 2.2 Participants

This study collected data from three participants: two osseointegrated participants with a transfemoral amputation (OI-TFA-1 and OI-TFA-2) and one able-bodied participant (AB-1). The participants’ characteristics are reported in Table 1.

**Table 1:** The participants’ characteristics.

Characteristics	OI-TFA-1	OI-TFA-2	AB-1
Gender	M	F	F
Side of amputation	Right	Left	-
Cause of amputation	Trauma	Trauma	-
Height (m)	1.91	1.695	1.74
Weight (kg)	78.3	59.3	68.5
Prosthetic knee	Ottobock C-Leg (microcontrolled)	Ottobock C-Leg (microcontrolled)	-
Prosthetic ankle	n/a (passive)	Ottobock Trias (passive)	-
Walking speed (m/sec)	0.527	1.111	0.888

## 3 Musculoskeletal models

This section presents the musculoskeletal model of a healthy subject provided in the OpenSim 4.1 environment, and explains the development of a generic musculoskeletal model of people with an unilateral transfemoral amputation wearing a generic osseointegrated prosthesis in OpenSim.

### 3.1 Musculoskeletal model—Healthy subject

**Fig 2:**
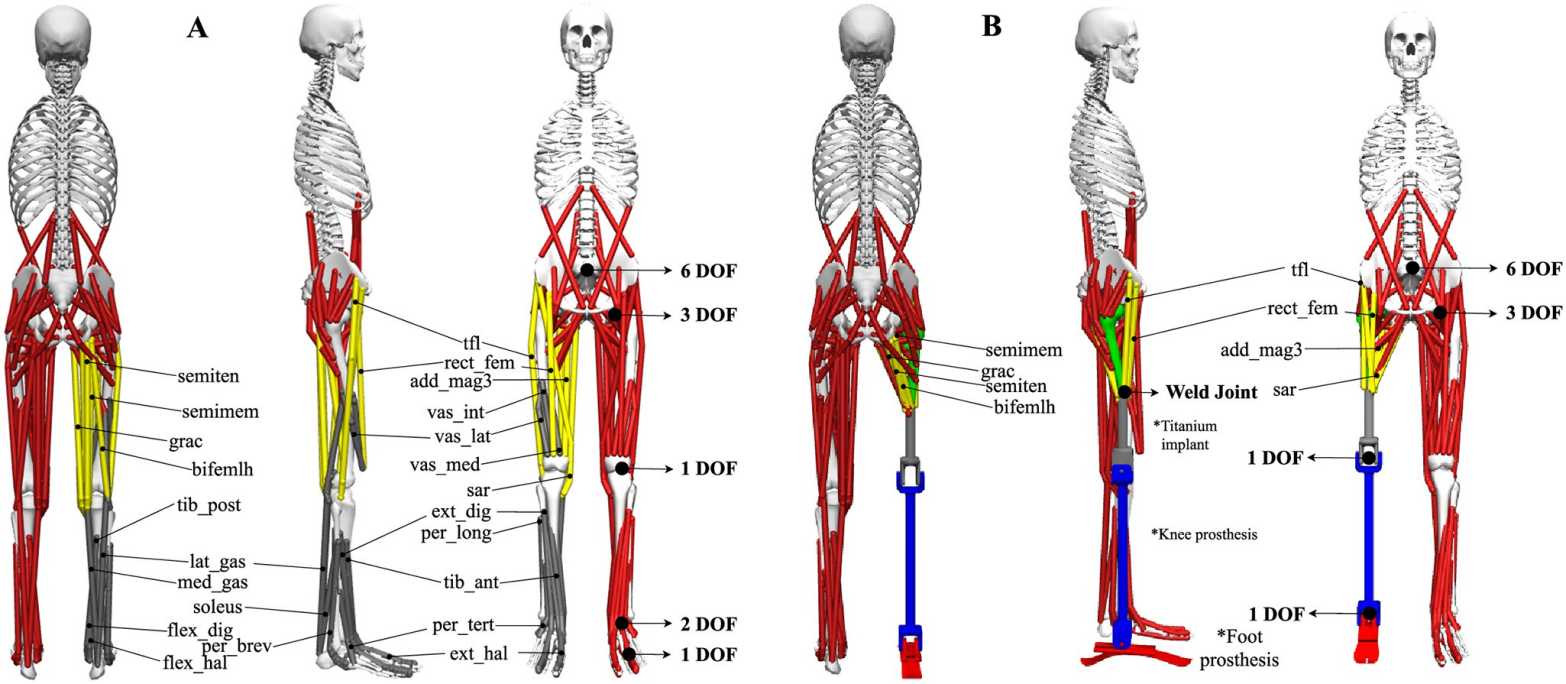
(A) Generic OpenSim musculoskeletal model of a healthy subject with 23 DOF and 92 musculotendon units (gait2392_simbody.osim). (B) The generic OpenSim musculoskeletal model of people with an unilateral transfemoral amputation wearing a generic prosthesis with 19 DOF, 76 musculotendon units, and two actuators, as proposed in this study. The muscles in yellow are transected and anchored to the transected femur bone, while the muscles in grey are removed in the proposed model.

OpenSim provides a generic lower-extremity musculoskeletal model (i.e., *Gait2392*, gait2392_simbody.osim), for performing kinematic and dynamic analyses of healthy subjects. The model consists of 23 degrees of freedom (DOFs) and 92 musculotendon units [23], as shown in Fig 2A. Table 2 summarizes the 23 DOFs (i.e., 3 for the torso, 6 for the pelvis, 3 for each hip joint, 1 for each knee joint, 1 for each ankle joint, and 2 for each foot). It should be noted that, the two DOFs of the feet (i.e., subtalar_angle and mtp_angle) have been locked in our simulations for a fair comparison between the different subjects. The 92 musculotendon units are symmetrically distributed over the left and right sides (i.e., 43 for the movement of each leg and 3 for the lumbar support at each side). The muscles are modelled as Hill-type muscles [24].

**Table 2:** Joint description of the OpenSim healthy subject model *Gait2392*.

Body	DOFs	Joint type	Joint names
Torso	3	Ball and socket	lumbar_extension
			lumbar_bending
			lumbar_rotation
Pelvis	6	Ball and socket + Translational	pelvis_tilt
			pelvis_list
			pelvis_rotation
			pelvis_tx
			pelvis_ty
			pelvis_tz
Hips (left and right)	3+3	Ball and socket	hip_flexion
			hip_adduction
			hip_rotation
Knees (left and right)	1+1	Revolute	knee_angle (flexion/extension)
Ankle (left and right)	1+1	Revolute	ankle_angle (dorsiflexion/plantarflexion)
Feet (left and right)	2+2	2x Revolute	subtalar_angle
			mtp_angle

### 3.2 Musculoskeletal model—Generic osseointegrated transfemoral amputee with a generic prosthesis

This study proposes to modify the musculoskeletal model *Gait2392* to create a generic musculoskeletal model of people with an unilateral transfemoral amputation wearing a generic osseointegrated prosthesis. The model consists of 19 DOFs and 76 musculotendon units, as shown in Fig 2B. Table 3 summarizes the 19 DOFs (i.e., 3 for the torso, 6 for the pelvis, 3 for each hip joint, 1 for each knee joint, 1 for each ankle joint). The muscles are modelled as Hill-type muscles [24]. Without loss of generality and for the sake of brevity, the proposed osseointegrated transfemoral model is described hereafter for the right-side amputation. The procedure is the same for the left leg.

**Table 3:** Joint description of the proposed OpenSim transfemoral amputee model.

Body	DOFs	Joint type	Joint names
Torso	3	Ball and socket	lumbar_extension
			lumbar_bending
			lumbar_rotation
Pelvis	6	Ball and socket + Translational	pelvis_tilt
			pelvis_list
			pelvis_rotation
			pelvis_tx
			pelvis_ty
			pelvis_tz
Hip (left and right)	3+3	Ball and socket	hip_flexion
			hip_adduction
			hip_rotation
Knee (left and right)	1+1	Revolute	knee_angle (flexion/extension)
Ankle (left and right)	1+1	Revolute	ankle_angle (dorsiflexion/plantarflexion)

**Fig 3:**
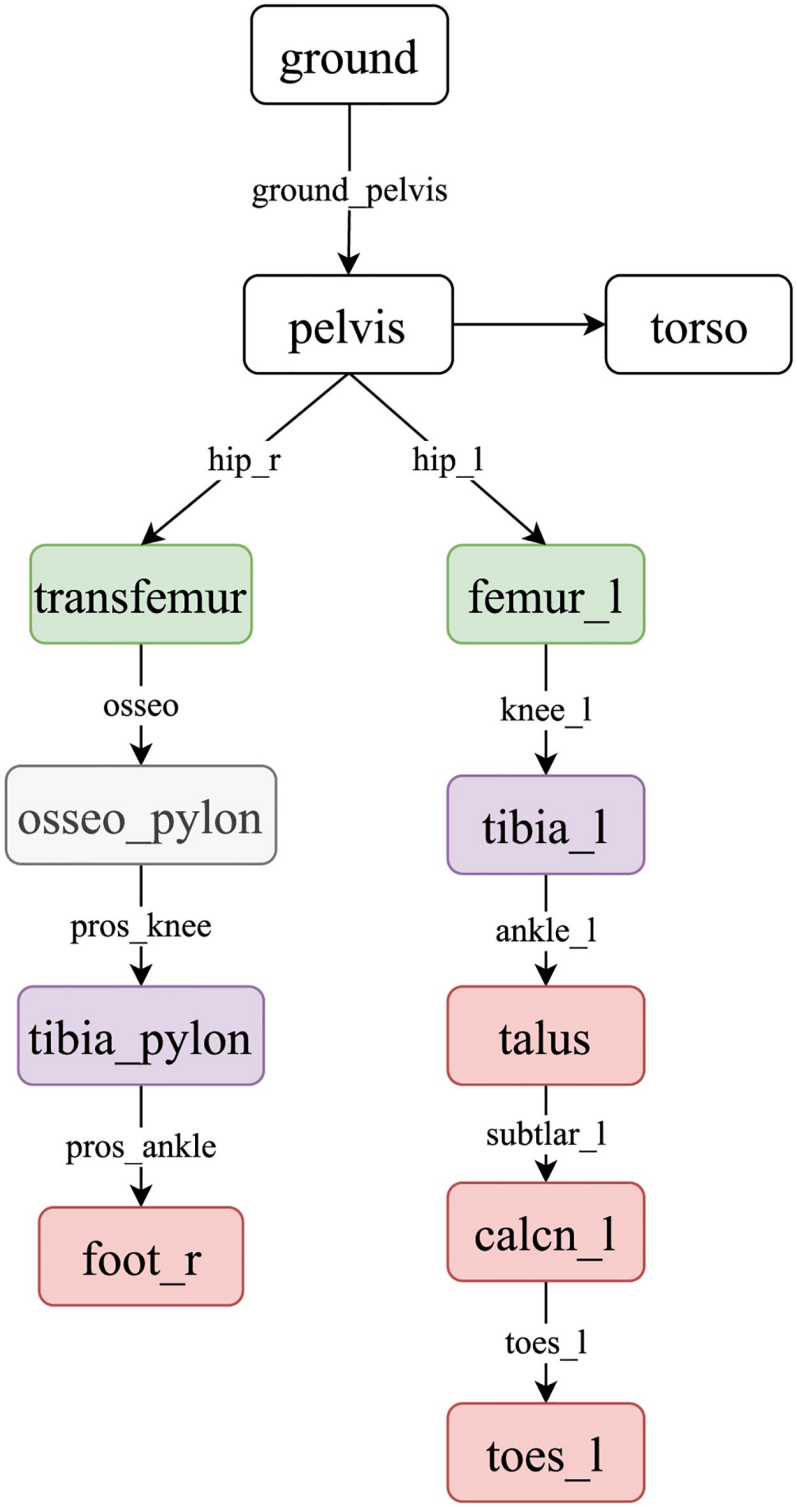
Model topology of osseointegrated transfemoral amputee model in OpenSim. The subtlar_l and mtp_l are marked in red to represent the locked DOF.

Fig 3 shows the topology of the model proposed in this study. The pelvis body is attached to the ground with 6 DOFs. The hip joints are connected to the pelvis along with the upper extremity as one solid mass, i.e., the torso. The body segments of each limb are given the same color in the figure to represent similarity with the contralateral limb. For instance, it can be observed that, for the right limb, a weld joint is introduced between the transected femur bone to represent the osseointegrated pylon (i.e., osseo_pylon). The tibia_pylon represents the transfemoral prosthesis, which is attached to the knee (i.e., the pros_knee) and to the ankle (i.e., the pros_ankle) joints. The foot_r represents the prosthetic foot. Here ‘pros’ indicates the prosthesis in short. To simplify the model for analysis, the subtalar and metatarsal joints at the ankle and toes of the contralateral limb in the model are locked at their neutral position as the main focus of the study is on the sagittal plane.

The proposed model, specified for both left- and right-side amputation, is made open-source and provided as support material to this study.

#### 3.2.1 Skeletal model and prosthesis

The right femur of the *Gait2392* model has been transected in half to have a generic femur length of 0.247 m. The osseointegrated pylon is designed as a combination of a femur implant (inside the femur bone), a locking screw, and a connector for the prosthetic attachment, as in the actual osseointegrated implants [25]. The total length of the osseointegrated implant is 0.205 m and the chosen material is the titanium alloy Ti6Al4Nb. The tibia pylon of the prosthesis has a length of 0.43 m, which includes the knee joint and the attachment for the ankle joint. The centers of the knee and ankle joints are matched with the centers of rotations of the knee and ankle joints of the contralateral limb for symmetry, as it would have been performed by a prosthetist.

The osseointegrated implant, tibia pylon, and foot are modeled using Solidworks (Dassault Systémes SolidWorks Corporation, USA, www.solidworks.com) and exported as .*stl* files to be imported in OpenSim. Table 4 details the mass values of the different body segments of the model as well as the inertial properties, based on the used materials. As the femur bone has been transected, its inertial properties and center of mass have been recalculated using the bone density of 350 *kg*/*m*^3^ [26]. For the prosthesis, the average mass of commercial microprocessor-controlled prosthetic knees and ankles-feet available in the market were used to weigh the tibia pylon and the foot, respectively. The CAD design shown in this model is for visualization purposes [27], and does not contribute to the computation apart from the geometric properties mentioned above.

**Table 4:** The mass of the body segments, center of mass (x, y, z), and their inertial properties (*I*_*xx*_, *I*_*yy*_, *I*_*zz*_) of the generic unscaled osseointegrated transfemoral model.

Body	Mass (kg)	Center of Mass (m)	Inertia [*I*_*xx*_, *I*_*yy*_, *I*_*zz*_] (kg m^2^)
Transected femur	4.65	0, -0.07, 0	0.02269, 0.0026, 0.02392
Osseointegrated implant	0.35	0, -0.133, 0	0.0013, 0.0001, 0.0013
Tibia pylon	1.4	0, -0.213, 0	0.0324, 0.0005, 0.03215
Foot	0.8	0.0186, -0.0213, 0	0.00036, 0.0025, 0.0025

#### 3.2.2 Muscles

The colors of the muscles used in Fig 2 are to be noted. The sixteen grey muscles in Fig 2A are removed from the *Gait2392* model because they are the amputated lost muscles (i.e., vastus medialis, vastus intermedius, vastus lateralis, gastrocnemius medialis, gastrocnemius lateralis, soleus, tibialis posterior, flexor digitorum longus, flexor hallucius longus, tibialis anterior, peroneus brevis, peroneus longus, peroneus tertius, extensor digitorum longus, extensor hallucius longus, biceps femoris short head). The eight (bi-articular) muscles in yellow are re-attached and anchored to the transected femur bone and, eventually, disabled while performing simulations (i.e., semimembranosus, semitendinosus, biceps femoris long head, sartorius, adductor magnus, tensor fascia latae, gracilis, and rectus femoris). The reasons for disabling these muscles is mainly due to the lack of clinical evidence of their contribution to the gait in the literature, and to the lack of detailed physical information that could be taken into account in a patient-specific model.

## 4 Method

This section presents the overall workflow in OpenSim to perform the inverse kinematic and the inverse/forward dynamic simulations of the proposed musculoskeletal model during level ground walking. Even if this workflow is a standard procedure in OpenSim, some of the OpenSim settings had to be modified to account for the specific information regarding the model and, more precisely, the transected femur bone and muscles, the prosthesis, and the two ideal actuators at the knee and ankle joints of the prosthesis.

Starting from the experimental data of the three participants (i.e., OI-TFA-1, OI-TFA-2, AB-1), the musculoskeletal model is made subject-specific. The experimental data are, then, used as input for the inverse kinematic and inverse dynamic simulations. By analyzing the results of the inverse kinematic/dynamic simulations on the three subject-specific models and, specifically, by analyzing the simulated hip/knee/ankle joints angles and knee/ankle joints torques, the correctness of the model is evaluated in Section 5. Finally, the model is validated by showing that the knee/ankle joints angles are closely matching the experimental data of the three participants.

To perform the forward dynamic simulation, the Computed Muscle Control of OpenSim is modified to account for the model. By analyzing the results of the forward dynamic simulations of the three subject-specific models and, specifically, by analyzing the simulated muscle forces and hip/ankle joints power, the correctness of the modified Computed Muscle Control is evaluated in Section 5. Finally, the novel optimal control architecture (i.e., the modified CMC) is validated by showing that the simulated muscle forces are in accordance with the biomechanical data of healthy subjects and compatible with observations made on transfemoral amputees subjects. For further analysis, another forward dynamic simulation is performed to observe the muscle contributions of an osseointegrated transfemoral amputee when a healthy gait would to be tracked thanks to a prosthesis with ideal actuators, that could be provided to people with an osseointegrated unilateral transfemoral amputation.

**Fig 4:**
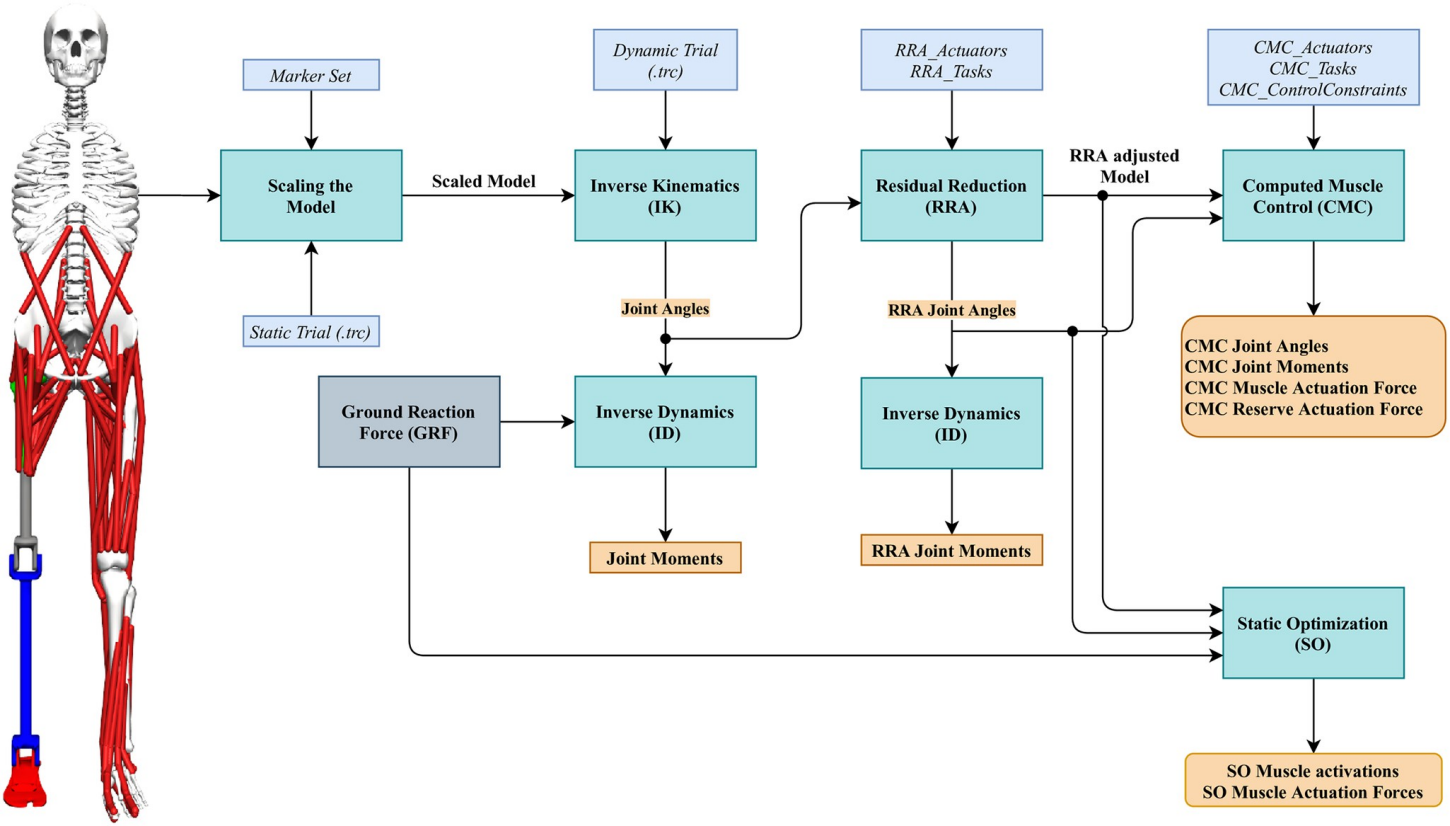
The workflow to perform the forward dynamics simulation in OpenSim of the proposed. The unscaled model is shown on the left. The cyan boxes are the toolbox available in OpenSim. The blue boxes are the input and settings files. The orange boxes are the generated outputs. The grey box contains the ground reaction forces.

The overall workflow is shown in Fig 4 and is described in details in the following subsections.

### 4.1 Data conversion (from .*c3d* to OpenSim files)

The motion data captured using the Vicon system can be exported as .*c3d* files, a standard biomechanics file format. However, OpenSim recognizes .*trc* format for the marker positions and .*mot* format for the forces data. Therefore, the MOtoNMS toolbox (MOtion data elaboration TOolbox for NeuroMusculoSkeletal applications, Matlab, Mathworks, Natick, MA, USA, http://www.mathworks.com) has been used to perform the data processing and conversion to OpenSim compatible files, as well as to save the reference frames and general settings in separate .*xml* files [28]. From the collected data, trials that contained only partial steps on the force plates have been removed.

### 4.2 Scaling the model

To make it subject-specific, the generic osseointegrated transfemoral amputee model is scaled by using the 28 virtual markers placed on the model itself and the actual markers in the experimental data. Specifically, the error between the virtual and the actual markers is iteratively reduced till it is minimized to an acceptable value, as per the guidelines in [29]. In the static scaling, a root-mean-square-error (RMSE) of 1.01 cm, 0.95 cm, and 0.87 cm for the OI-TFA-1, OI-TFA-2, and AB-1 models has been obtained, respectively.

For the scaling process, the bony landmark markers are given a higher static weight to better match the position of the virtual and the actual markers. For example, the markers on the lateral knee and the ankle are given a static weight of 5, while the markers on the upper body are set to 1. However, these values can be iteratively adjusted until an acceptable value is achieved, as per the guidelines in [29]. To scale the different segments of the model, marker pairs are used, e.g., the thigh can be scaled by using the lateral knee marker and the hip joint center, which can be computed with the Vicon gait plugin [22].

It is important to highlight that the scaling of the model is a very critical process for performing the simulations. Therefore, the photos and videos taken during the experimental data collection have to be extensively used to accurately place the virtual markers on the model.

### 4.3 Inverse kinematics

After scaling the model, the positions of the actual marker from the motion capture data (.*trc* files) are given as input to the OpenSim Inverse Kinematics toolbox to compute the generalized positions (i.e., the joint angles) of the DOFs of the model.

In the setup files for the OpenSim Inverse Kinematics toolbox, the markers that identify the segments of model (especially for the thigh and the tibia) are given a higher weight than the bony landmark markers. For the lower-extremity markers, the maximum marker error and the RMSE should be less than 2–4 cm and less than 2 cm, respectively [30]. This process is iterated along with the scaling if the errors are above the thresholds. Furthermore, it is useful, and therefore common practice, to compare the simulated gait in the OpenSim visualizer with the experimental video captured during the data collection.

### 4.4 Inverse dynamics

The OpenSim Inverse Dynamics toolbox uses the mass information from the scaled model, the generalized positions (from the inverse kinematics results), the generalized velocities and accelerations (computed from the generalized positions) and the ground reaction forces (GRFs, collected from the force plates during the experiments) to solve the equations of motion for the unknown generalized forces/torques at each DOF of the model. The GRFs are plugged into the model by defining the points of applications (on both the calcaneus and the prosthetic foot) and the forces/torque, as provided in the force plate data (.*mot* files).

During the inverse dynamic simulations, the scaling of the model has been rechecked. A RMSE of 0.72 cm, 0.88 cm, and 1.31 cm for the OI-TFA-1, OI-TFA-2, and AB-1 models has been obtained, respectively. As the RMSE are still as per the guidelines in [29], the performed scaling in Section 4.2 has been kept.

### 4.5 Residual Reduction Algorithm (RRA)

To minimize the effects of modeling and of the marker data processing and, specifically, to make the results of the inverse dynamics more consistent with respect to the GRFs, the OpenSim Residual Reduction Algorithm (RRA) toolbox is used to adjust model’s mass. During this iterative process, the residuals (i.e., the non-physical compensatory forces automatically added by OpenSim to run the inverse dynamics) are reduced and, finally, minimized. In this study, the RRA brought to a total mass change of 2.106 kg, −0.197 kg, and 1.181 kg for the OI-TFA1, OI-TFA2, and AB-1 models, respectively. Upon these adaptations, the average residual forces applied to the pelvis (i.e., hand-of-god actuators) were minimized to less than 1% of the center of mass height times the magnitude of the measured net external forces, as per the guidelines in [29].

### 4.6 Modified Static Optimization (mSO)

The OpenSim Static Optimization (SO) toolbox solves the equations of motion for the unknown generalized forces/torques at each DOF of the adapted (after the RRA) model as the results of the muscle forces. A modified Static Optimization (mSO) is proposed in this study that takes into account the two actuators at the knee and ankle joints of the prosthesis. Specifically, the mSO computes the unknown generalized forces/torques *τ*_*j*_ while satisfying the condition on the force-length-velocity property:

$$\begin{array}{c} \sum_{m=1}^{n}\left[a_{m}f\left(F_{m}^{0},l_{m},v_{m}\right)\right]r_{m,j}=\tau_{j} \end{array}$$ (1)

and while minimizing the objective function (i.e., the sum of the squared muscle activations and of the squared torques of the two actuators at the ankle and knee joints):

$$\begin{array}{c} J=\sum_{m=1}^{n}{\left(a_{m}\right)}^{2}+\sum_{m=1}^{2}{\left(a_{act}\right)}^{2} \end{array}$$ (2)

where *n* is the the number of muscles (i.e., 76 in the proposed model), *a*_*m*_ the activation level of the muscles, *a*_*act*_ the activations of the actuators at the ankle and knee joints of the prosthesis, $F_{m}^{0}$, *l*_*m*_, and *v*_*m*_ the maximum isometric force, length, and shortening velocity of the muscle, respectively; $f(F_{m}^{0},l_{m},v_{m})$ the active fiber force [12, 31], *r*_*m*,*j*_ the moment arm about the j^*th*^ joint axis.

In the mSO, the OpenSim reserve actuators (associated to the prosthetic knee and ankle joints) are enabled to compute the knee and ankle torques along with the muscle forces. This step was performed to investigate whether it was feasible to perform a forward dynamic simulation with the proposed model.

### 4.7 Modified Computed Muscle Control (mCMC)

To perform the forward dynamic simulation of the model, a modified Computed Muscle Control (mCMC) of the OpenSim CMC is proposed in this study that takes into account the two actuators at the knee and ankle joints of the prosthesis.

**Fig 5:**
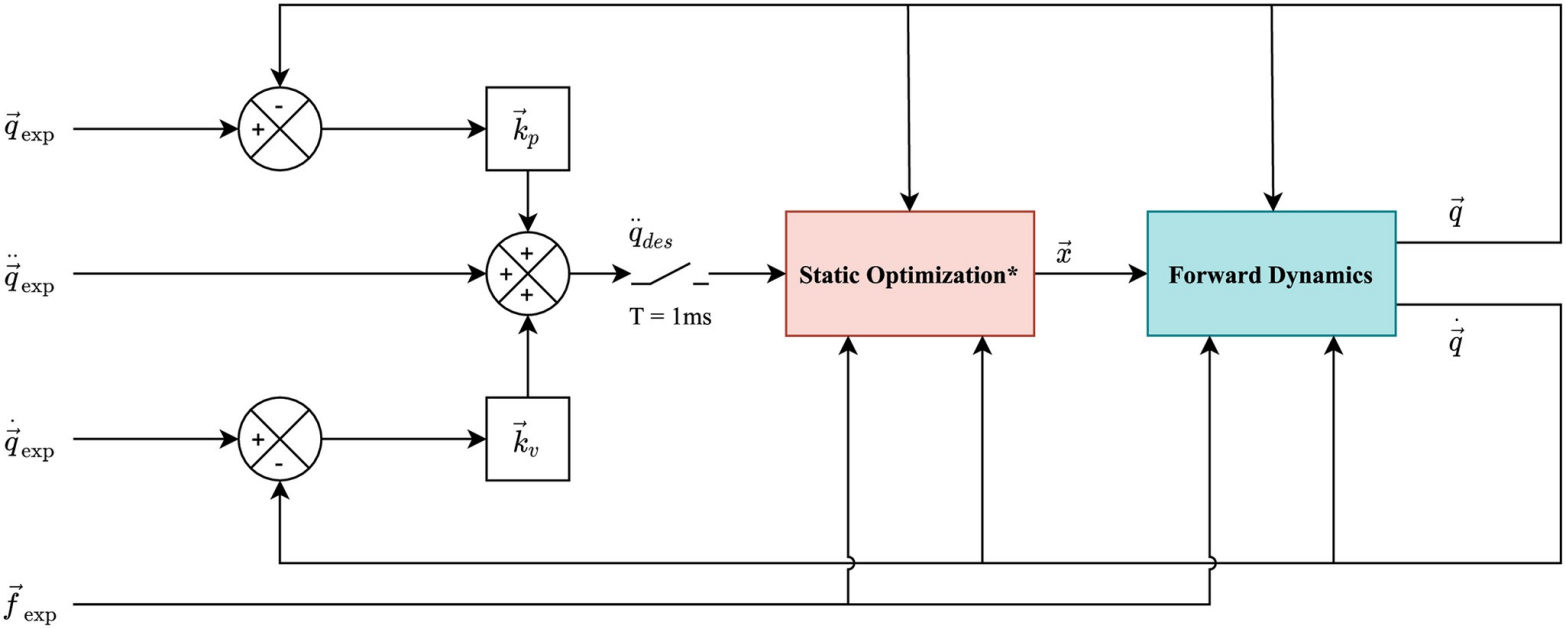
The Opensim CMC is modified to perform the forward dynamic simulation of the proposed model. The control architecture relies on an optimal control in which the cost function accounts for the muscles excitations and the two actuators at the knee and ankle joints of the prosthesis.

The mCMC computes the muscle excitations and the actuators’ torques that drive the generalized positions of the model towards the desired kinematic trajectory, as in the experimental data. As shown in Fig 5, the CMC uses a combination of a proportional-derivative feedback control (with control gains ${\vec{k}}_{p}$ and ${\vec{k}}_{v}$, respectively) and the static optimization on the error between the experimental and the model accelerations (${\ddot{\vec{q}}}_{exp}$ and $\ddot{\vec{q}}$), velocities (${\dot{\vec{q}}}_{exp}$ and $\dot{\vec{q}}$), and positions (${\vec{q}}_{exp}$ and $\vec{q}$), i.e.:

$$\begin{array}{c} {\ddot{\vec{q}}}_{des}\left(t+T\right)={\ddot{\vec{q}}}_{exp}\left(t+T\right)+{\vec{k}}_{v}\left[{\dot{\vec{q}}}_{exp}\left(t\right)-\dot{\vec{q}}\left(t\right)\right]+{\vec{k}}_{p}\left[{\vec{q}}_{exp}\left(t\right)-\vec{q}\left(t\right)\right] \end{array}$$ (3)

The mCMC takes as input three settings files and the tracking data (see Fig 4). The *CMC_Actuators* file defines the list of reserve actuators used to support the simulation to compensate for additional forces to maintain the stability of the model. For instance, during the gait cycle, if the muscles or actuators in the model cannot provide sufficient forces to compensate the GRF, the reserve actuators are triggered. The mCMC minimizes the contribution of the reserve actuators, i.e., minimizes the following objective function:

$$\begin{array}{c} J\left(x,\ddot{q},w\right)=\sum_{i=1}^{n_{M}}{\left({\frac{a_{i}}{f_{max\_iso}}}_{i}\right)}^{2}+\sum_{j=1}^{n_{R}}{\left(\frac{x_{j}}{\tau_{max_{j}}}\right)}^{2}+\sum_{k=1}^{n_{k}}w_{k}{\left({\ddot{\vec{q}}}_{k_{\text{des}}}-{\ddot{\vec{q}}}_{k}\right)}^{2} \end{array}$$ (4)

where, *n*_*M*_ is the number of muscles, *a*_*i*_ the activation of the *i*^*th*^ muscle within the range of [0.2, 1], *f*_*max*_*iso*__*i*_ the maximum isometric force of the *i*^*th*^ muscle, *n*_*R*_ the number of reserve actuators, *x*_*j*_ the control value within the range of [0, 1], *τ*_*max*__*j*_ the maximum torque that the actuator can apply, *n*_*k*_ the number of joints in the model, *w*_*k*_ the weights for *k*^*th*^ coordinate, ${\ddot{\vec{q}}}_{k_{\text{des}}}$ and ${\ddot{\vec{q}}}_{k}$ are the desired and model accelerations, respectively.

Similarly, the other two setting files *CMC_Tasks* and *CMC_Control_Constraints* define the weights of the joints that are to be tracked and the gains for the proportional-derivative controllers for each actuator in the model.

The main changes in the mCMC with respect to the OpenSim CMC toolbox are:

- In the OpenSim CMC, all the muscles use *k*_*p*_ and *k*_*v*_ as 100 and 20, respectively. However, for the proposed mCMC, as it is the combination of muscles and two ideal actuators at the knee and ankle joints, the *k*_*p*_ and *k*_*v*_ for the actuators have been set to 1000 and 90, respectively. These values were obtained after observing the behavior of actuators and imposing the dynamics of a critically damped system.
- The OpenSim CMC has been exploited to recruit actuators to provide assistive forces and to study its effects of designing exoskeletons [19, 20]. Similarly, the proposed mCMC has to recruit the knee and ankle actuators whose optimal torques have been set to 1 MNm. The rest of the reserve actuators are given the default minimum torques of 1 Nm. The role of optimal torques is conveyed in the Eq 4, where *τ*_*max*__*j*_ is used to recruit the actuators of the prosthesis as they have the magnitude in the order of MN. This ensures that the cost function *J* is penalized less for the prosthetic actuators than the other reserve actuators. At the end of each simulation, the residual forces are checked to see if any reserve actuators are triggered on the joints powered by the muscles.

**Fig 6:**
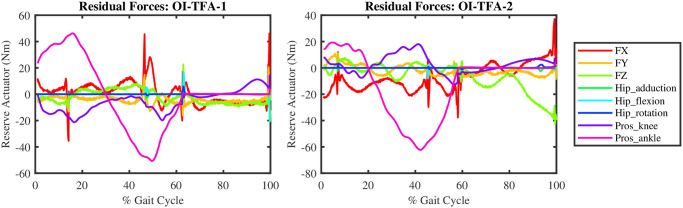
The residual forces generated by the (reserve) actuators at some joints of the subject-specific models of the two osseointegrated participants (OI-TFA-1 and OI-TFA-2).

Fig 6 reports the residual forces generated by the reserve actuators at some of the joints of the model when the forward dynamic simulation by means of the mCMC is performed on the data of the two osseointegrated participants (OI-TFA-1 and OI-TFA-2) on the corresponding subject-specific models. It is possible to notice that the (reserve) actuators are only triggered for the prosthetic knee and ankle actuators (Pros_knee and Pros_ankle) while the other residual actuators (FX, FY, FZ for the hand-of-god actuators at the pelvis, and the Hip_adduction, Hip_flexion, Hip_rotation) are negligible. This implies that the combination of muscles forces and actuators’ torques is such that a forward dynamic simulation can be performed and, therefore, that the method presented in this section is sound and can be further analysed.

## 5 Results and discussion

In this section, the simulation results obtained using the method described in Section 4 are presented and discussed. The results are also shown in the video that is provided as support material to this study.

### 5.1 Inverse kinematic and inverse dynamics analysis

This subsection shows the results of the inverse kinematic and inverse dynamic simulations of the three subject-specific models (i.e., OI-TFA-1, OI-TFA-2, AB-1) during level ground walking, which have been obtained by using the OpenSim Inverse Kinematics and Inverse Dynamics toolboxes, respectively, on the experimental data of the three participants.

#### 5.1.1 Joint angles

**Fig 7:**
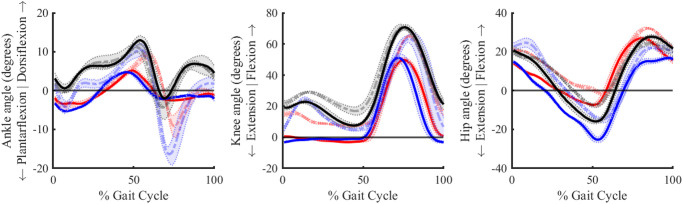
Angles in the sagittal plane for the ankle, knee, and hip joints during level ground walking as a result of the inverse kinematic simulation. Red, blue, black color represents the participant OI-TFA1, OI-TFA-2, and AB-1, respectively. The continuous thick lines show the mean joint angles of the amputated leg for the participants with transfemoral amputation, and of the right leg for the healthy participant. The dashed thick lines show the contralateral limb. The shaded region represents the standard deviation of the joint angles over five gait cycles.

Fig 7 shows the average (over five gait cycles) angles for the ankle, knee, and hip joints in the sagittal plane for both legs (OI-TFA-1 in red, OI-TFA-2 in blue, and AB-1 in black) during level-ground walking. The continuous thick lines show the mean joint angles of the amputated leg for the participants with transfemoral amputation, and of the right leg for the healthy participant. The dashed thick lines show the contralateral limb. The shaded region represents the standard deviation of the joint angles over five gait cycles. From the figure, it is possible to notice that:

- The passive ankle-foot prosthesis cannot provide an active plantarflexion during the late-stance (60–70%) of the gait cycle, which is in agreement with the general behavior of passive ankle-foot prostheses [32].
- The behavior of the ankle and knee joint angles are in agreement with the gait data of people with transfemoral amputation of using a (microcontrolled) prosthetic knee and a passive ankle-foot, as reported in [32].
- The contralateral ankle of both people with transfemoral amputation shows an excessive plantarflexion (12.65° and 18.56° for OI-TFA-1 and OI-TFA-2, respectively). This behavior is in agreement with the study in [32], where people with transfemoral amputation need to provide extra toe-clearance when walking with passive prosthetic feet.
- The (microcontrolled) prosthetic knee cannot provide the stance-flexion (which, on the contrary, is visible at their contralateral limb), and is fully extended throughout the stance phase. This behavior is in agreement with the kinematics of microcontrolled prosthetic knees [32].
- The behavior of the three joints of the healthy participant are in agreement with the healthy participant data during level ground walking [12]. However, there is an offset in the hip flexion because, while in the *Gait2392* model the neutral position is 0°, in clinical studies, the neutral position of the hip flexion is considered at ∼12–13°.

It is possible to conclude that the inverse kinematic simulation of the proposed model is in accordance with the kinematics of people with transfemoral amputation wearing a passive ankle-foot prosthesis [17] and a microcontrolled prosthetic knee [32], herein validating the correctness of the proposed model.

#### 5.1.2 Joint torque vs. joint angles

**Fig 8:**
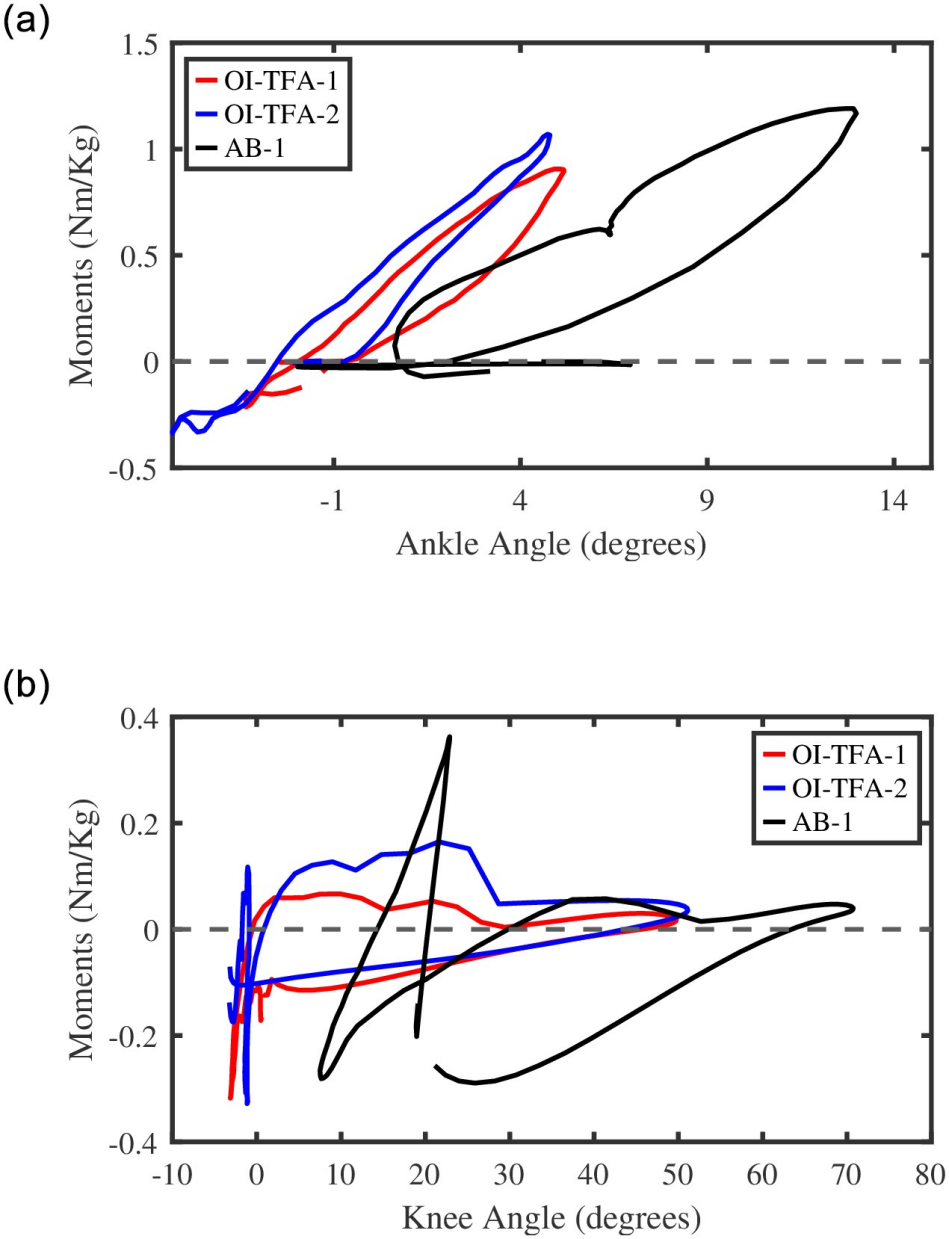
(left) The relationship between the normalized ankle joint moment and ankle joint angle and (prosthetic limb for participants OI-TFA1 and OI-TFA2, the right leg for AB-1) as the results of the inverse dynamic simulation. (right) The relationship between the normalized knee joint moment and knee joint angle (the prosthetic limb for participants OI-TFA1 and OI-TFA2, and right leg for AB-1) as the results of the inverse dynamic simulation.

Fig 8 (left) shows the relationship between the angle and the torque of the ankle joints (normalized on body-weight) of the three subject-specific models (prosthetic ankle for the transfemoral amputees and right ankle for the healthy participant). It is possible to note that, while for the healthy participant the foot can perform a plantarflexion, this is not possible for the two people wearing the passive ankle-foot prosthesis. This is indeed a limitation of passive ankle-foot prosthesis, which cannot provide the complete range of motion with respect to the required torque for level-ground walking and it is correctly captured by the proposed model [32].

Fig 8 (right) shows the relationship between the angle and the torque of the knee joints (normalized on body-weight) of the three subject-specific models (prosthetic knee for the transfemoral amputees and right knee for the healthy participant). It is possible to note that the (microcontrolled) prosthetic knee damps the impact forces at heel strike (∼0°). Also, the prosthetic knee is able to support the swing phase (∼0° − 50°), where the angle/torque relation follows the healthy participant profile without, however, guaranteeing the same range of motion. Moreover, while the knee of the healthy participant shows a smooth absorption of the impact forces during the complete stance (∼10° − 25°), the prosthetic knee shows a sharp peak throughout the stance phase (∼0°), when it is fully extended.

It is possible to conclude that the inverse dynamic simulation of the proposed model is in accordance with the dynamics of people with transfemoral amputation wearing a passive ankle-foot prosthesis and a microcontrolled prosthetic knee [32], herein completing the validation of the proposed model.

### 5.2 Forward dynamic analysis

This subsection shows the results of the forward dynamic simulation of the subject-specific models (i.e., OI-TFA-1, OI-TFA-2, AB-1) during level ground walking, which have been obtained by using the mCMC on the experimental data of the three participants. This simulation allows an analysis of the gait of people with transfemoral amputation (OI-TFA-1 and OI-TFA-2) and a comparison with a healthy gait (AB-1) in terms of movements (i.e., joint kinematics) and muscles contributions (i.e., muscle excitation and muscle forces).

Moreover, this subsection shows the results of the forward dynamic simulation of the transfemoral model (scaled on the AB-1 participant) during level ground walking, which has been obtained by using the mCMC on the experimental data of the able-bodied participant (AB-1). This simulation, hereafter labeled as OI-TFA-AB-1, allows an analysis of how the muscle contribution of an osseointegrated transfemoral amputee would be when a healthy gait has to be tracked but with a prosthesis using ideal actuators.

#### 5.2.1 Joint angles

**Fig 9:**
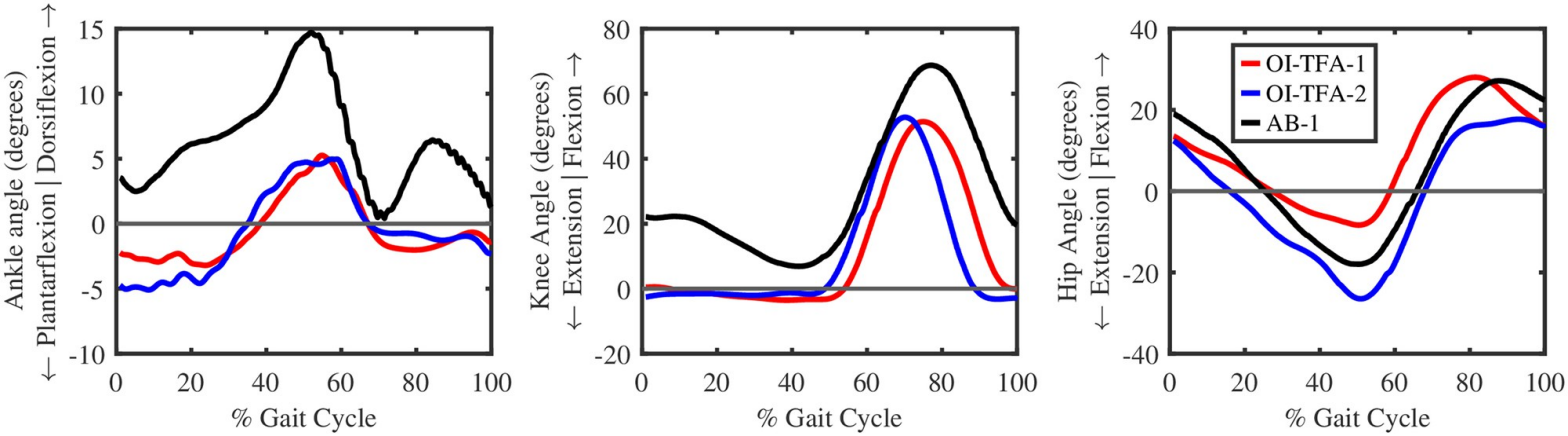
Angles in the sagittal plane for the ankle, knee, and hip joints during level ground walking as the result of the modified computed muscle control simulation for one gait cycle for the contralateral leg of OI-TFA-1 (red) and OI-TFA-2 (blue), and for the right leg of AB-1 (black).

Fig 9 shows the joint angles for the ankle, knee, and hip in the sagittal plane for the contralateral leg for OI-TFA-1 and OI-TFA-2 (in red and blue, respectively) and for the right leg of AB-1 (in black). The average position tracking errors are overall very low, i.e., 0.188° and 0.144° for the ankle joint of OI-TFA-1 and OI-TFA-2, respectively, 0.02° and 0.035° for the knee joint of OI-TFA-1 and OI-TFA-2, respectively, and 0.104° and 0.136° for the hip joint of OI-TFA-1 and OI-TFA-2, respectively. This good accuracy was obtained by using the fast target formulation of static optimization in performing the mCMC and a 1 ms look-up time, which allowed the muscles to follow their first-order activation dynamics [24] while at the same time to not conflicting with the ideal actuators that have an instantaneous dynamics.

It is possible to conclude that the proposed model together with the proposed optimal control architecture (i.e., the mCMC) can reproduce the kinematics of both healthy people [12] and of people with transfemoral amputation wearing a passive ankle-foot prosthesis and a microcontrolled prosthetic knee [32].

#### 5.2.2 Muscle forces

**Fig 10:**
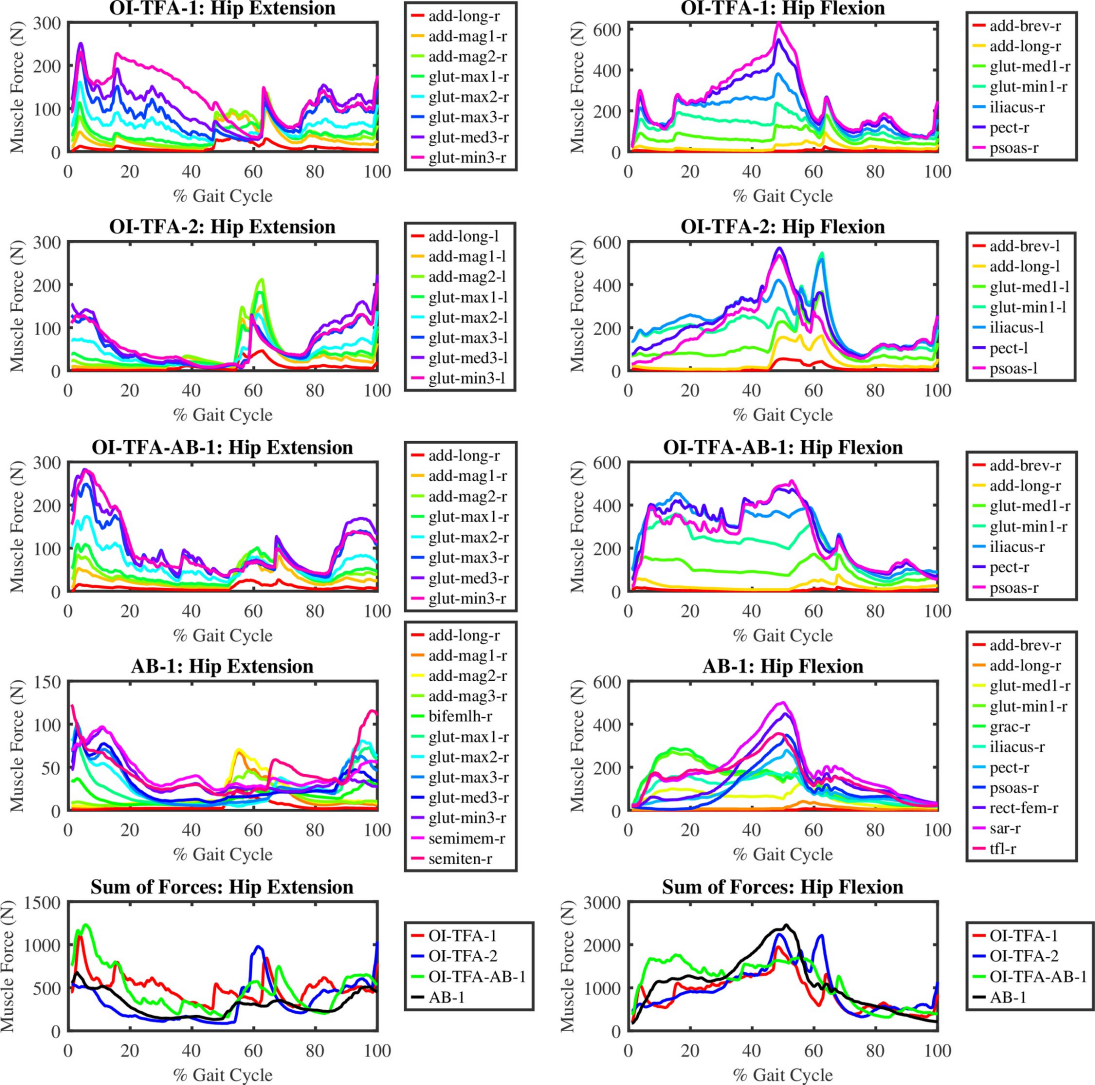
Muscle forces for the hip flexion and extension of the amputated leg during a gait cycle. For the AB-1, the results from the right leg are shown.

Fig 10 shows the muscle forces for the hip flexion and extension during one gait cycle of the subject-specific models as the results of the mCMC on the experimental data of the three participants. It can be noticed that:

- AB-1 uses the least muscle force for the hip extension when compared to OI-TFA-1 and OI-TFA-2. The hip extensors are triggered the most for OI-TFA-1, OI-TFA-2, and OI-TFA-AB-1 during the heel strike and toe-off. This behavior is not observed for AB-1. However, the muscle recruitment pattern remains the same as the adductor longus, gluteus minimus, and gluteus medius contribute majorly to the hip extension.
- The peak force at the toe-off for the hip extension is similar in OI-TFA-1 and OI-TFA-2. Interestingly, the OI-TFA-AB-1 and AB-1 show comparatively lower peak force generation at toe-off. This could result from the active force generation of the ideal actuators as it mimics the able body gait for level ground walking compared to the passive prosthetic foot. The semimembranosus and semitendinosus muscles are active during the hip extension for AB-1 which are missing in OI-TFA-1 and OI-TFA-2. This could be the reason for distributing the forces to the gluteus muscles in OI-TFA-1 and OI-TFA-2.
- The hip flexion is wider during the mid-stance and at toe-off for all the models. However, the pelvic muscles (e.g., psoas, iliacus, and pectineus muscle) are triggered mainly for OI-TFA-AB-1 and are active throughout the stance phase to compensate for the role of the missing sartorius, tensor fascia latae, and gracilis muscles. Furthermore, it aids in matching the kinematics for the hip flexion during the healthy gait.
- The gracilis and gluteus minimus provide gait stability from heel strike till mid-stance for AB-1, and the rest of the hip flexors are engaged after the mid-stance to flex the hip towards the toe-off.

From the figure, it is possible to conclude that the muscles forces generated during the forward dynamic simulation for the hip extension/flexion are compatible with the muscle forces of able body subjects [32], and they are sound with respect to the analysis done on transfemoral amputee subjects [33] even if muscle data are not provided in the literature for this class of subjects.

**Fig 11:**
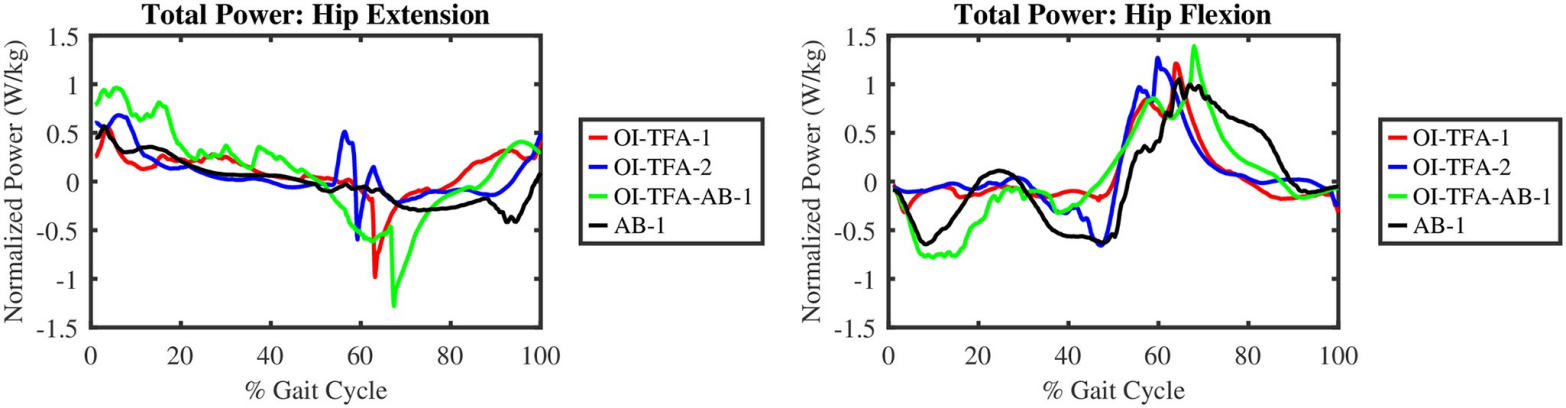
Total power produced by hip flexors and extensors of the amputated leg during a gait cycle. For the AB-1, the results from the right leg are shown.

Fig 11 shows the hip power, normalized on body weight, along one gait cycle. Form the figure, it is possible to compute the total energy consumption for the hip extensors, i.e., 8.99, 6.75, 11.04, and −0.78 Joules/kg for OI-TFA-1, OI-TFA-2, OI-TFA-AB-1, and AB-1 respectively. It is evident that, as expected, due to the lack of muscles at the hip, the energy required for the hip extension is much higher to perform a healthy gait. In fact, while AB-1 absorbs energy to perform the gait, the other models release energy to perform the gait. From the figure, it is possible to compute also the total energy consumption for the hip flexion, i.e., 6.02, 7.04, 2.88, and 3.26 Joules/kg for OI-TFA-1, OI-TFA-2, OI-TFA-AB-1, and AB-1 respectively. It is evident that the actuators at the ankle and knee joint provide assistance to minimize the effort to sustain the gait.

**Fig 12:**
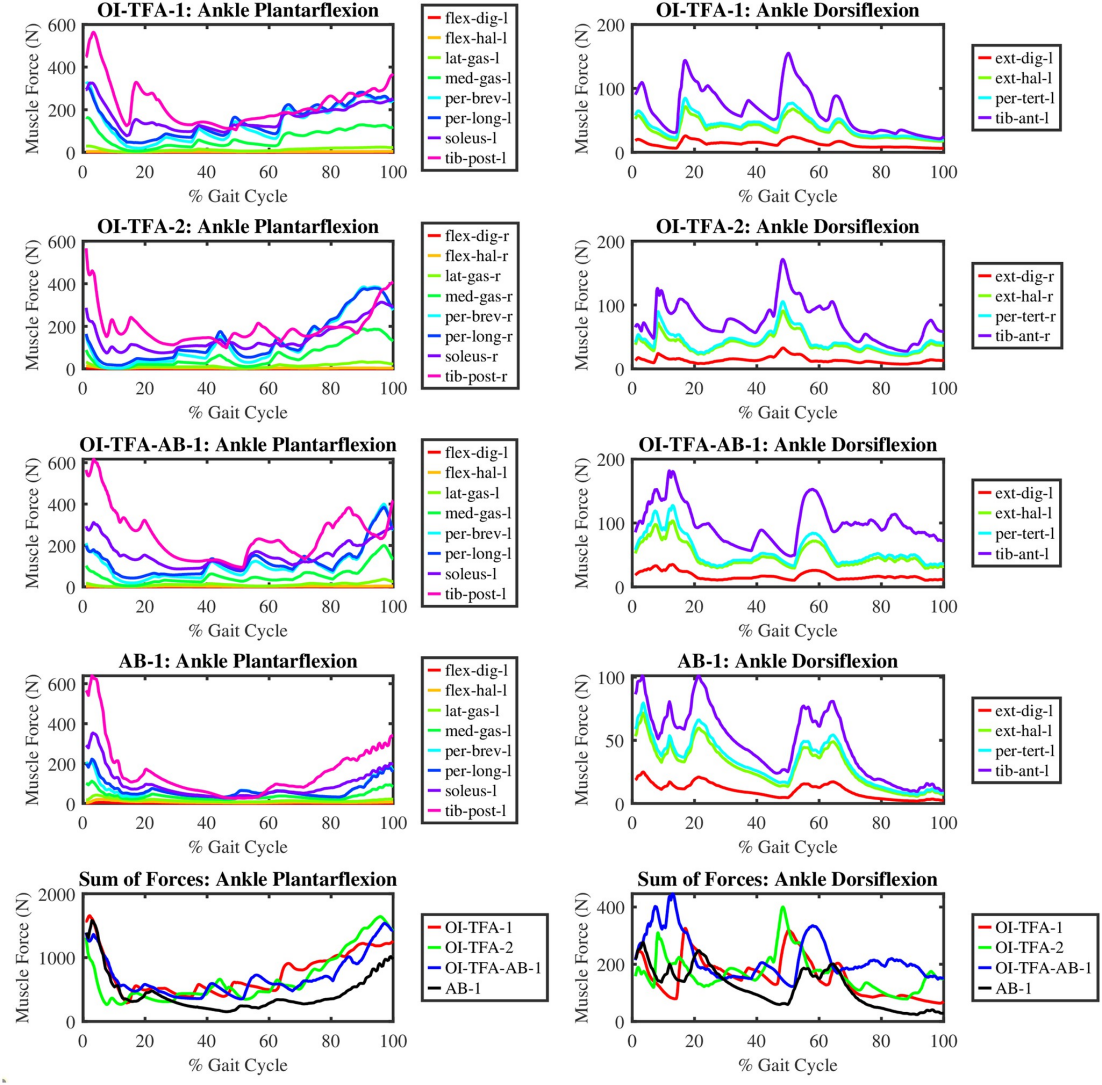
Muscle forces for the ankle plantarflexion and dorsiflexion of the contralateral limb during a gait cycle. For the AB-1, the results from the right leg are shown.

Fig 12 shows the muscle forces for the ankle plantarflexion and dorsiflexion during one gait cycle of the three subject-specific models as the results of the mCMC on the experimental data. It can be noticed that:

- The ankle plantarflexors provide extra toe-clearance for the OI-TFA-1 and OI-TFA-1 compared to the more efficient AB-1. The soleus, tibialis posterior, and peroneus muscles are the most active during the swing phase (60–100%) of the prosthetic leg as they perform an excessive plantarflexion. The gastrocnemius (i.e., a biarticular muscle that is responsible for plantarflexion but mostly for the stability of the gait) is also seen to perform as expected.
- For the ankle dorsiflexion, the tibialis anterior is one of the major contributors, as observed in all the models consistently. In the double stance phase (50–70% of the gait cycle), the dorsiflexors are triggered to accept the heel strike of the contralateral limb and perform the stance. Even though the dorsiflexors are triggered at toe-off, the dominance is shown by the plantarflexors to provide toe-clearance. It can also be observed that the muscle force pattern for OI-TFA-1 and OI-TFA-2 are similar in terms of force generation. This shows a good consistency in the results of the simulations on the proposed model.

From the figure, it is possible to conclude that the muscles forces generated during the the forward dynamic simulation for the ankle plantarflexion and dorsiflexion are compatible with the muscle forces of able body subjects [32], and they are sound with respect to the analysis done on transfemoral amputee subjects [33] even if muscle data are not provided in the literature for this class of subjects.

**Fig 13:**
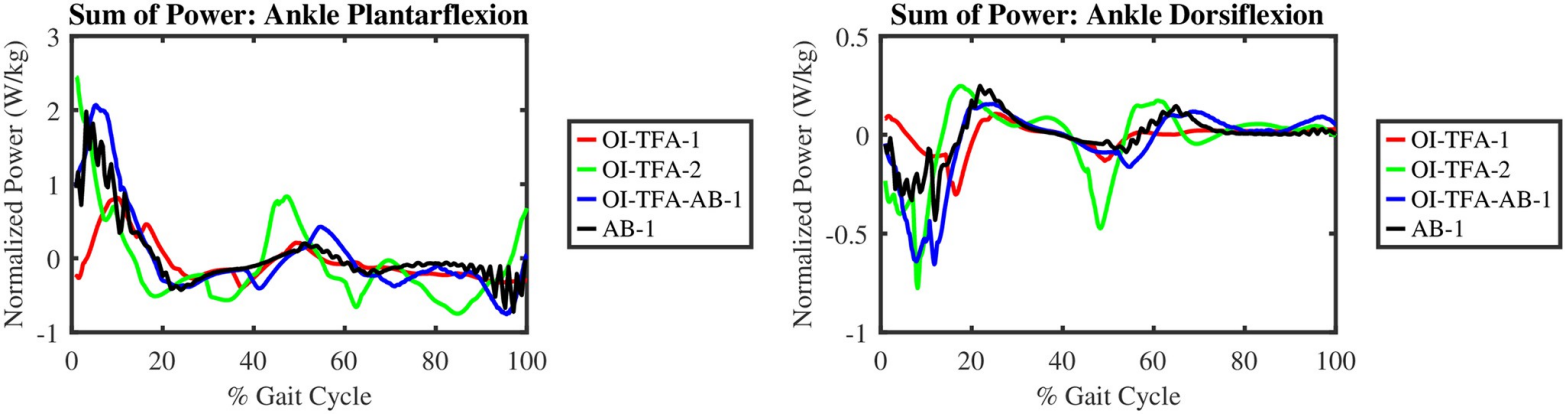
Total power produced by the ankle plantarflexors and dorsiflexors of the contralateral limb during a gait cycle. For the AB-1, the results from the right leg are shown.

Fig 13 shows the ankle power, normalized on body weight, along one gait cycle. Form the figure, it is possible to compute the total energy consumption for the ankle plantarflexion, i.e., −4.91, −7.30, 3.50, and 3.60 Joules/kg for OI-TFA-1, OI-TFA-2, OI-TFA-AB-1, and AB-1, respectively. It is evident that OI-TFA-1 and OI-TFA-2 absorb more energy than OI-TFA-AB-1 and AB-1 because the transfemoral amputee gait has a longer stance phase than the healthy gait. From the figure, it is possible to compute also the total energy consumption for the ankle dorsiflexors, i.e., −0.67, −2.0, −3.61 and −0.3 Joules/kg for OI-TFA-1, OI-TFA-2, OI-TFA-AB-1, and AB-1, respectively. It is evident that OI-TFA-2 and OI-TFA-AB-1 show similar power consumption because of similar preferred gait speed (as reported in Table 1), and because OI-TFA-AB-1 has also to accommodate for the impact at heel strike and to compensate for the lost muscles.

#### 5.2.3 Limitations and outlook to the future

Previous work has focused on a physics-based musculoskeletal model of a socket-user amputee wearing a transtibial prosthesis in OpenSim [17], and on its validation via inverse kinematic simulations [18]. As contribution to the current state of the art, this study focuses on the physics-based musculoskeletal model of people with an osseointegrated transfemoral prosthesis in OpenSim, and on its validation via inverse kinematic, inverse dynamic, and forward dynamic simulations. While the inverse kinematic and dynamic simulations fully show a good match between the simulated hip/knee/ankle joints and the experimental data (see Figs 7 and 9) and, therefore, a validation of the proposed lower-extremity musculoskeletal model, the forward dynamic simulations show muscle forces that are compatible with healthy [32] and amputee subjects [33] This makes the validation of the proposed mCMC only qualitative within this study. Future work will focus on the development of an even more accurate digital twin of the participants, which will include experimental data on the muscle activity.

## 6 Conclusion

In this study, we developed a generic lower-extremity musculoskeletal model of people with an osseointegrated unilateral transfemoral amputation wearing a generic transfemoral prosthesis in OpenSim. Moreover, we proposed a modification to the OpenSim computed muscle control to be able to perform the forward dynamic simulation of such a model and, specifically, to account for the ideal actuators at the knee and ankle joints of the prosthesis. The model has been qualitative validated by observing in the simulation results on three different participants, the kinematics, the kinetics as well as the muscle forces, which present patterns typical of transfemoral amputees wearing passive prostheses and of healthy participant.

The model is a powerful tool that can be used by the scientific community to perform biomechanical analysis of people with a transfemoral amputation wearing an osseointegrated prosthesis. Specifically, even if left for future work, the model can be used to understand which kind of passive prostheses could improve the gait while minimizing specific performance metrics, or which kind of controller should be implemented on the actuators of a micro-controller/powered prosthesis to better support the user and to further enhance the gait.

## Supporting information

S1 Data The OpenSim musculoskeletal model of a person with an osseointegrated unilateral transfemoral amputation wearing a generic transfemoral prosthesis, specified for both left- and right-side amputation, is provided as support material. It is available at https://simtk.org/projects/oi-tfp-model.
(ZIP)

S1 Video A video of the OpenSim forward dynamic simulation as presented in this study is provided as support material. (MP4)
